# 25, 50 and 75 Years Ago

**DOI:** 10.1111/ans.70145

**Published:** 2025-04-23

**Authors:** Julian A. Smith

**Affiliations:** ^1^ Department of Surgery Monash University Melbourne Victoria Australia

**Keywords:** healthcare

## 25 Years Ago

### L. Watterson, B. Flanagan, B. Donovan, and B. Robinson, “Anaesthetic Simulators: Training for the Broader Healthcare Profession,” *ANZ Journal of Surgery* 70 (2000): 735–737

The use of high‐fidelity patient simulators for training healthcare professionals has increased rapidly in recent years. Approximately 150 simulation training centres operate internationally. Australasia has acquired four centres since 1997. A large component of simulator‐based training is experiential. Participants manage clinical scenarios on lifelike computer‐controlled mannikins within realistic clinical environments. Afterwards, they actively reflect upon the experience, an exercise that is facilitated by observation of a video replay of the event. This approach to training promotes a consideration of broader issues which can influence clinical practice and patient out‐ comes. This has particular relevance to emergencies. Here, events that are by nature infrequent and unscheduled can be addressed in a controlled fashion, in an environment that is supportive and separated from actual patients. A broad range of skills can be addressed with this resource. Of key importance are situational management and team effectiveness skills. Deficiencies with respect to these ‘non‐clinical’ skills are being increasingly identified for their contribution to preventable adverse events within the healthcare environment. Multidisciplinary operation‐room team training has the potential to address these issues as they relate to the perioperative environment.

### P. Crowe, P. Harris, and J. Ham, “Teaching on the Run: Teaching Skills for Surgical Trainees,” *ANZ Journal of Surgery* 70 (2000): 227–230

Increasing recognition of the need for training in teaching skills for clinical teachers has coincided with data that registrars and residents conduct much ‘on the job’ teaching as part of their routine work. While attention has been devoted to training consultants, support for the teaching role of the junior staff has been relatively neglected. The aim of the present report is to describe the teaching experiences of surgical registrars and the impact of a registrar teaching workshop. A half‐day programme combining presentation and discussion of surgical teaching with practical skills sessions was designed for surgical registrars at Prince of Wales Hospital. The programme included observation and feedback of brief teaching simulations at the bedside of volunteer patients to newly commenced clinical students, and small group sessions on clinic and operating theatre teaching. A pre‐workshop questionnaire sought information about the registrars' own teaching, and a survey 3 months after the workshop determined if any changes to teaching practice had occurred. The registrars were generally moderately to very confident with their teaching ability but more than 75% felt that they were more confident after the workshop. Only three of 39 registrars had received any instruction aimed at improving their teaching skills, yet 34/39 had taught either on the ward, in the clinics or in the operating room. Follow‐up after 3 months revealed that most registrars were enjoying their teaching tasks more, and half had increased their teaching since the workshop and began discussing teaching with their surgical colleagues. The present project demonstrates that relatively brief interventions focused on skill development may enhance the confidence and enjoyment of junior clinical teachers and increase the frequency of ‘teaching on the run’.

## 50 Years Ago

### R. D. Marshall and G. P. Gay, “Hiatal Hernia Repair by Posterior Gastropexy,” *ANZ Journal of Surgery* 45 (1975): 376–380

**FIGURE 1:**
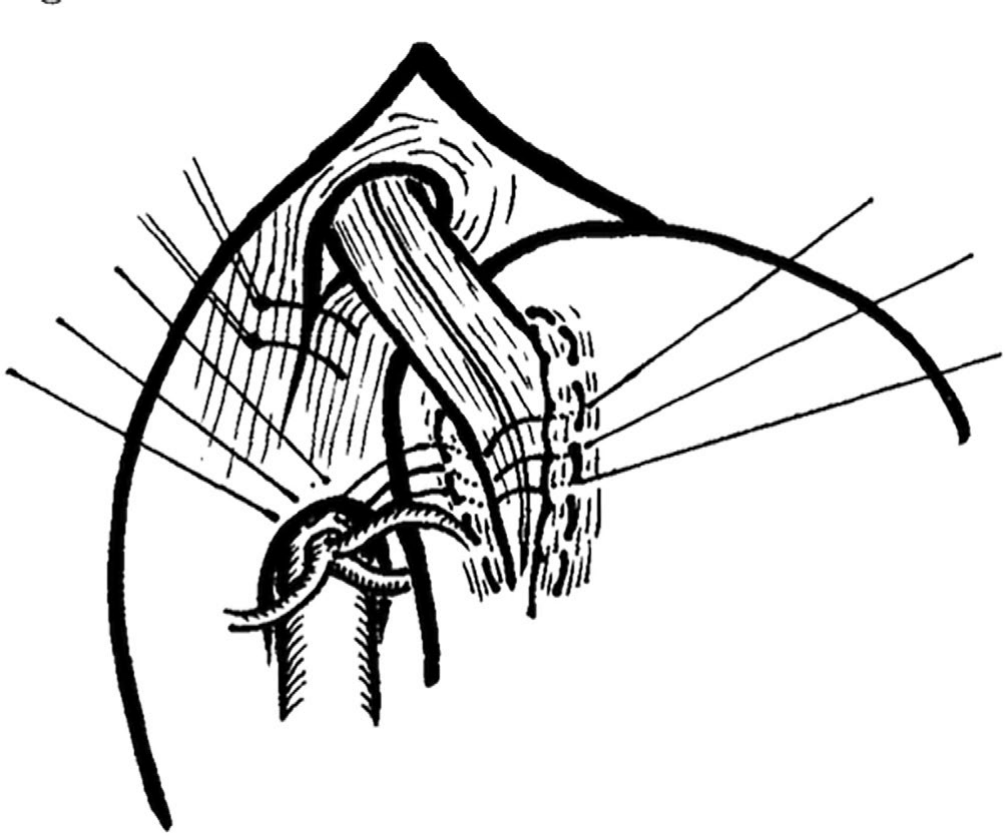
The limbs of the esophageal hiatus are sutured together behind the oesophagus, and the incisural muscle sling is sutured to the median arcuate ligament.

Posterior gastropexy was used in the repair of 77 hiatal hernias from 1970 to 1975, after the fashion of Lucius D. Hill of Seattle. The operations were performed by one author and reviewed by the other. The rationale and technique (Figure 1) of the operation are discussed. Ten patients were lost to follow‐up. Fifty‐six patients were greatly improved or cured, 10 were improved but still had some symptoms, while one was unimproved. We conclude that posterior gastropexy is an effective, simple, and safe operation for the treatment of reflux associated with hiatal hernia.

### R. Rowland, R. Hecker, R. J. Fitch, G. E. Gibson, R. Willing, and W. L. Jennings, “Early Gastric Carcinoma,” *ANZ Journal of Surgery* 45 (1975): 349–354

Seven cases of early gastric carcinoma are reported. This condition by definition refers to a carcinoma confined to the mucosa and submucosa only. The definition depends upon the depth of spread and not the surface area covered by turnour or the period over which it is believed to have been present. Each patient had a history indistinguishable from one of benign ulceration. All seven patients had barium meal studies, and in only two was the possibility of malignancy raised. In the other five patients, the ulcers demonstrated radiologically were considered benign. Fibreoptic endoscopy was done in six cases; in three, the appearances were suspicious of malignancy, and in the remaining three, the lesions appeared benign. Biopsy specimens taken at endoscopy contained carcinomatous tissue in all six cases. The patients all had partial gastrectomy, and no recurrence has yet been found. The longest follow‐up period is 36 months.

## 75 Years Ago

### F. W. Niesche, “Carcinoma of the Lower Oesophagus and Upper Stomach,” *ANZ Journal of Surgery* 19 (1950): 221–231

Since little has appeared in the Australian literature concerning surgery of the lower oesophagus and upper stomach, it is of interest to record a series of cases and, in particular, to stress the pitfalls and mistakes which have occurred. Records of 13 patients submitted to total gastrectomy or cardio‐oesophagectomy are presented. Eight patients survived the operation, one dying at the end of 6 months from acute intestinal obstruction. Seven patients are still living, four having survived for a period of at least 12 months. A plea for early diagnosis has been made because all these patients had advanced malignant disease and some were poor risk. The operability rate has been extended as far as possible. With earlier diagnosis and increasing experience, it is felt that the mortality can be lowered. These extensive operations may be considered justifiable if the patient can live in comparative comfort for a year or more.

